# Neuropsychiatry as a paradigm propelling neurology and psychiatry into the future

**DOI:** 10.1192/bjo.2024.864

**Published:** 2025-03-03

**Authors:** Jay A. Salpekar, Marco Mula, Niruj Agrawal, Kenneth R. Kaufman

**Affiliations:** Departments of Psychiatry and Neurology, Kennedy Krieger Institute, Johns Hopkins University School of Medicine, USA; Department of Neurology, St George’s University Hospital and Neurosciences Clinical Academic Group, St George’s University of London, UK; Department of Neuropsychiatry, St George’s Hospital, London, UK; South West London and St Georges Mental Health NHS Trust, London, UK; Department of Neurology, St George’s University of London, UK; Departments of Psychiatry, Neurology and Anesthesiology, Rutgers Robert Wood Johnson Medical School, Rutgers University, USA; Department of Psychological Medicine, School of Academic Psychiatry, Institute of Psychiatry, Psychology and Neuroscience, King’s College London, UK; Department of Psychiatry, University of Oxford, UK

**Keywords:** Behaviour, diagnosis and classification, neurology, neuropsychiatry, psychiatry

## Abstract

Neurology and psychiatry have long been divided as subspecialities of medicine. However, the symptom overlap in central nervous system illness is unmistakable. Medical science has evolved, necessitating a neuropsychiatric approach that is more comprehensive. This editorial briefly outlines the history of neurology and psychiatry and the movement towards a new paradigm.


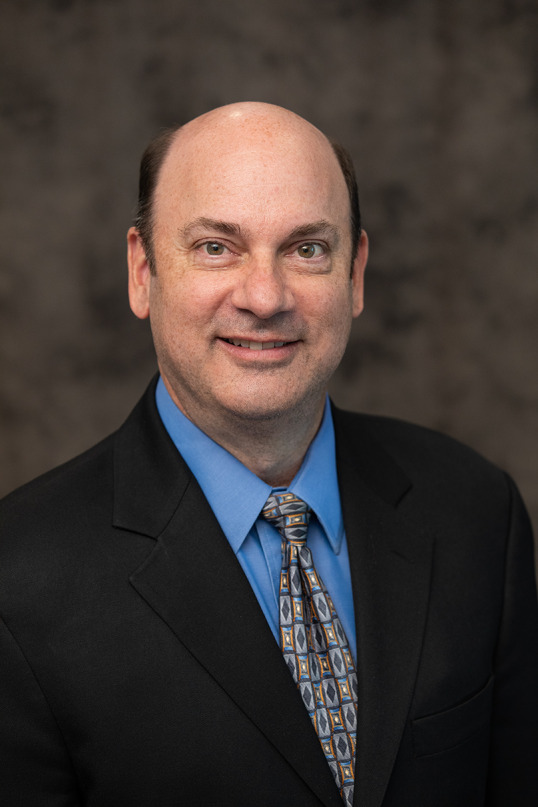
The status quo in 2024 is still that neurology and psychiatry are two separate specialities, even as the heterogeneity within each of those fields is enormous. Within neurology, some are specialised in epilepsy, some in neurodegenerative cognitive disorders, and some in abnormal movements among many others. Psychiatry is even broader, with many subspecialities based on age groups, specific settings such as forensics or general hospital liaison or based on specific conditions such as eating disorders. While many practitioners emphasise psychotherapeutic interventions of varying types, others at least in part may de-emphasise talk therapy to the degree they are referred to in the field as ‘prescribers’.^1,2^

Oddly, this de-emphasis is occurring despite the fact that many successful adjunctive non-medical treatments have been developed in recent years. Psychological approaches in particular, such as short-term targeted therapies and dialectical approaches, may complement biologically oriented psychiatric practices. Standard of care for epilepsy neurosurgery incorporates neuropsychology consultation with psychometric testing as part of the neurological assessment with resultant better definition of risk profiles and improved surgical outcomes. While these examples are encouraging, coordination of psychiatry with neurology has been elusive. While a team approach is optimal, this is too often not feasible. For psychiatry, psychotherapy and pharmacology are not commonly integrated, with multiple clinicians promoting nuanced treatments, typically without communicating with one another. While it is beyond the scope of this discourse to assess the degree to which psychologically minded treatment should be integrated into practices of psychiatry or neurology, or indeed any medical speciality, the reality is that psychiatry and neurology in most centres have remained largely isolated from one another.

The emergent field of neuropsychiatry may be the natural compensatory response to this bifurcation. At the very least, it represents one grassroots attempt to seek a comprehensive approach to a wide swath of medical illness. Patients commonly complain about the siloed approach to medicine. They want their neurologists to help them manage the depression that may be a constituent part of their epilepsy.^3^ They are frustrated by psychiatrists who refuse to treat behaviour problems if an active neurological condition is also present.^4^ Similarly, psychiatrists are frustrated by neurologists and fellow psychiatrists not addressing the double stigma associated with mental illness and neurological problems such as epilepsy.^5^ Further, specialists may have limited knowledge of iatrogenic effects of drugs used by both neurologists and psychiatrists which complicate medication adherence.^6,7^ An unfortunate truth is that patients and families have reverted to desperate solutions to counter a lack of unified thinking by physicians. Perhaps a significant example of this frustration has been the long resistance of the medical community in considering cannabinoids as therapeutics for palliative care or for neuropsychiatric conditions including epilepsy, dementia or autism spectrum disorder.^8–11^

The present day then is a terrific time for a thematic series on neuropsychiatry. Not only will the articles in this thematic series show the fascinating conceptual overlap that is present, but in doing so, a necessary and revitalised paradigmatic blend of neurology and psychiatry will be expressed. That comprehensive sensibility may be a modern phenomenon, but the concept has roots in antiquity. For most of the history of medicine, neurology and psychiatry were not separated.^12^ Clinicians at the Salpetriere in the late 19th century had no such conceptual bifurcation. Sigmund Freud, the neurologist, proposed internal psychic phenomena as aetiological for ‘hysterical seizures’, that were induced famously by the neurologist, Jean Martin Charcot.^13^ Long before that, Hippocrates referred to the overlap between epilepsy and melancholia as different versions of the same underlying pathology.^14^ That approach was prescient and validates the significant psychiatric symptoms in many diseases considered exclusively ‘neurological’. Psychiatric symptoms such as anxiety or panic also overlap with many other medical disorders as well.^15^

Epilepsy is a robust example of the overlap and is well represented in the literature, but movement disorders, cerebrovascular events, autoimmune and neuroinflammatory conditions represent additional examples of illnesses that cannot be solely viewed as neurological or psychiatric. Movement disorders particularly have also long been viewed as requiring an interdisciplinary approach.^16^ Both fields place a heavy emphasis on mental status exams as informative for diagnostic evaluation. The ripple effects of central nervous system diseases are far reaching, and presenting or persisting symptoms may include inattention, misperceptions or affective lability along with more characteristic motor or sensory impairments. Ultimately, the pressure to consider psychiatric phenomena and neurological phenomena as interrelated is unavoidable. Considering how symptoms interact then becomes the task of medical care delivery, notably impacting healthcare funding.

While this notion may seem intuitive, it is curious that a neuropsychiatric way of thinking has not become conventional wisdom. Possibly the residue of history has upheld a counterproductive inertia. It is important to remember that neurology as a field was fraught with ambiguity, until lesion studies in the 19th century propelled its development as a more accepted and separate medical science. Epilepsy represents another such dichotomy as it had long been regarded as a psychiatric illness until the advent of electroencephalography in the 1920s and 1930s. Nothing changed about the illness except for a way to measure it objectively, regardless of the inherent imprecision. Reduction of ambiguity by physical measurement has reassured generations of physicians seeking ‘hard science’ underpinnings to illness, even though those measures may be arbitrary. Reliance on physical measurement has always been challenged by scientific limitations, whether in the calibration capacity of the measure or in the viewpoint of the measurer. Changing definitions of hypercholesterolemia, hypertension and the like should make clinicians take pause regarding dependence on ostensibly objective data science. A dependence on measurable data has unwittingly led some neurologists to treat the brain while neglecting behaviour.

Psychiatry has evolved even more markedly. Even from the denouement of psychoanalysis, conflicting heuristics have long been present in psychiatry. Emil Kraepelin and Sigmund Freud were contemporaries in the burgeoning field of psychiatry, but had significantly diverging views, one proposing biological aetiologies of mental illness and the other promoting psychological causes. Although psychoanalysis was appealing as a path to personal growth and improvement, the ambiguity of diagnosis and treatment did not contribute to its suitability as a medical science. Medical model psychiatry emergent from Kraepelin was ironically propelled by non-classical psychiatrists such as Eli Robins and Sam Guze, both of whom applied categorical criteria common in internal medicine to psychiatry.^17,18^ In the mid 20th century, they proceeded to establish diagnostic validity with family and twin studies, and also developed a consistent nomenclature for the disease course.^19^ Yet more importantly, this was a grassroots effort, a recognition that physicians could not well care for many patients with somatisation disorder on internal medicine wards. A prescient statement made by Dr Sam Guze in ‘Biological Psychiatry: is there any other kind’ is that ‘Psychiatrists, including those who accept the fundamental biological basis of psychiatry, cannot ignore culture, philosophy, ethics, and religion as they try to understand psychiatric disorders.’^20^ Biological approaches to psychiatry did not discount or seek to rule out other diseases or fields of study, but instead strove to incorporate them in a comprehensive model. Neuropsychiatry may be a modern and intuitive extension of this formulation.

Adding to the challenge of modernising heuristics is the fact that both neurology and psychiatry address the most complex aspects of human existence. Even beyond medicine, an age-old question of philosophers is how to define human consciousness. For physicians, typical physiological underpinnings of consciousness are challenging, let alone pathophysiological aspects. Understandings of typical and atypical capacities of consciousness have also evolved over time. Within the last few centuries of human history, introspective human consciousness has slowly but refreshingly become recognised as existent across races, genders and ages. The notion that cognitive superiority selectively existed for some human subgroups was conventional wisdom for much of human history, even as we know today how absurd that notion is.^21^ However, today’s bifurcation of neurologists treating only the brain and the body, and psychiatrists treating the mind and behaviour, may be equally erroneous. Incorrect assumptions in medicine delayed the understanding of such groundbreaking developments as the germ theory or anaesthesia. Although further research is required, we may be missing similar groundbreaking understanding such as with the potential role of anti-neural antibodies in the onset of psychiatric illness.^22^

Now then is the time for neuropsychiatry. It may be the natural state of neurology and psychiatry, which cannot possibly be separated, any more than the mind and body can be separated. Both are limited by billing structures or service delivery models embedded in modern medicine, but also by our nomenclature and by the human need to classify and simplify in order to maintain homeostasis, both physically and mentally. Neuropsychiatry may be a subspeciality of both psychiatry and neurology, or it may be the foundation of both fields. Practitioners would then need to be trained in both fields, and have a common language that underpins the discourse, much like a language being efficiently spoken by a native speaker as opposed to trying to extrapolate concepts into a second language. Training programmes can then teach new conceptualisations, ones that are broader and that incorporate more therapeutic strategies. Training may be the essence of developing a new conceptual approach, and in that way, the next generation of clinicians geared towards clinical neuroscience may speak a different language, at least in part. Regardless of the inertia of the status quo, the trend towards neuropsychiatry is clearly growing. Although specialisation will not go away, having both neurologists and psychiatrists embracing a neuropsychiatric paradigm will enhance a sensibility that will be more useful for patients, clinicians and researchers in the future.

## Data Availability

Data availability is not applicable to this article as no new data were created or analysed in this study.
